# The effect of social exclusion on bedtime procrastination among college students: the mediating role of psychological resilience and the moderating role of fear of missing out

**DOI:** 10.3389/fpsyg.2026.1868352

**Published:** 2026-09-03

**Authors:** Jianbo Wang, Zixuan Zhang, Jiale Zhao

**Affiliations:** School of Psychology and Mental Health, North China University of Science and Technology, Tangshan, China

**Keywords:** bedtime procrastination, college students, fear of missing out, psychological resilience, social exclusion

## Abstract

**Background:**

In contemporary society, health issues have garnered widespread attention, with sleep being recognized as a fundamental determinant of overall wellbeing. For college students, maintaining adequate, high-quality sleep is particularly imperative, given its critical role in academic performance and psychological adjustment. Accordingly, the present study aimed to examine the association between social exclusion and bedtime procrastination among Chinese undergraduates and to explore the mediating role of psychological resilience and the moderating role of fear of missing out (FoMO) in this relationship.

**Methods:**

A total of 1,050 undergraduate students from a university in China were recruited to participate in this study. Data were collected via an online survey, yielding 972 valid questionnaires (effective response rate = 92.6%). Data analysis was conducted using SPSS 27.0 and the PROCESS macro. Correlation analysis, mediation effect testing, and moderated mediation analyses were conducted to systematically evaluate the relationships among the core variables.

**Results:**

The empirical results revealed that (1) social exclusion was significantly and positively correlated with bedtime procrastination among college students; (2) psychological resilience partially mediated the relationship between social exclusion and bedtime procrastination; and (3) FoMO moderated the direct pathway between social exclusion and bedtime procrastination within the mediation model, indicating that the strength of this association varied across different levels of FoMO.

**Conclusion:**

Social exclusion serves as a salient correlate and risk-enhancing factor for bedtime procrastination among college students, demonstrating a significant positive linkage. Specifically, social exclusion may indirectly intensify individuals’ bedtime procrastination by undermining their psychological resilience. Moreover, FoMO acts as a moderating mechanism, suggesting that the susceptibility of socially excluded individuals to bedtime procrastination is contingent upon their level of FoMO.

## Introduction

With rapid societal development and the accelerating pace of life, college students are confronted with a wide range of pressures and challenges, all of which are often accompanied by a series of psychological and behavioral issues. Among these, bedtime procrastination is particularly prominent (Guo and Chen, 2025). In 2014, Kroese et al. first proposed the concept of bedtime procrastination, which refers to individuals’ failure to go to bed at the intended time despite the absence of external interference (Kroese et al., 2014a). To avoid conceptual confusion, clearly define the scope of the study, and specify the measurement tool, it is necessary to distinguish between bedtime procrastination and while-in-bed procrastination: While the latter refers to delaying sleep after already being in bed, the former specifically concerns the failure to initiate the bedtime transition at the intended time (Magalhães et al., 2020). The present study focuses exclusively on bedtime procrastination. According to Chinese sleep survey data, approximately 80% of college students habitually prepare for bed after 23:30, far exceeding the scientifically recommended schedule (He et al., 2023). A cross-sectional study involving 20,704 Chinese college students indicated that approximately 10.5% exhibited severe bedtime procrastination (Zhang et al., 2025). Bedtime procrastination directly leads to reduced sleep duration and poorer sleep quality, ultimately resulting in sleep insufficiency. In severe cases, it not only jeopardizes physical health but may also trigger psychological problems such as depression and anxiety, thereby exerting a significant negative impact on college students’ daily academic performance and life (Broström et al., 2017; Campbell and Bridges, 2023). Therefore, an in-depth investigation of the influencing factors and psychological mechanisms underlying bedtime procrastination is of great significance for reducing this behavior and for promoting individuals’ physical and mental health.

The causes of bedtime procrastination are complex and likely the result of multiple interacting factors. Among these factors, the impact of social exclusion warrants particular attention. Social exclusion refers to the social harm individuals experience when they are marginalized in interpersonal interactions and primarily includes rejection (e.g., discrimination, stigmatization, dehumanization, or microaggressions) and neglect (e.g., being forgotten, ignored, or refused eye contact) (Wang Y. et al., 2020). On the one hand, existing research indicates that social exclusion can trigger negative emotions and lead individuals to doubt their interpersonal skills and self-worth (Niu et al., 2023). In turn, these experiences may prompt individuals to engage in behaviors aimed at achieving emotional catharsis and obtaining a compensatory value—for instance, by watching short videos for pleasure, engaging in physical exercise, or completing planned tasks to perceive their self-value. Notably, these activities can often only be performed at night (Chung et al., 2020). On the other hand, according to ego depletion theory, individuals’ internal resources are limited; once these resources are excessively depleted, self-control over subsequent behaviors is weakened (Baumeister et al., 1998). Therefore, as a negative social experience, social exclusion consumes considerable self-resources. Particularly at night, individuals’ self-control capacity to resist temptation and delay gratification is further diminished. Consequently, they lose the executive capacity to adhere to their “should sleep” plan and are unable to resist seeking the aforementioned emotional and value compensation, even at the expense of sleep. Therefore, individuals affected by social exclusion are likely to exhibit higher levels of bedtime procrastination. Based on this, the present study proposes the following hypothesis: There is a significant positive association between social exclusion and bedtime procrastination among college students.

As an external contextual factor contributing to maladaptive behaviors, social exclusion not only directly influences individuals’ external behaviors but also indirectly affects individual behaviors by depleting certain internal psychological resources, such as psychological resilience. Psychological resilience refers to an individual’s capacity to recover strength in the face of adversity, encompassing the ability to adapt and restore functioning after experiencing stress, challenges, or failure (Wu G. et al., 2013). Huang et al. (2025), based on a survey of 5,153 college students, demonstrated that psychological resilience is negatively associated with bedtime procrastination. According to the stress-buffering model (Cohen and Wills, 1985), psychological resilience, as a critical internal buffer resource, can help individuals resist the negative effects of stressors. When individuals are adversely affected by negative events, high levels of psychological resilience can serve as an appropriate buffering mechanism to reduce bedtime procrastination. Conversely, experiencing social exclusion has been found to undermine individuals’ psychological resilience (Yu et al., 2025), thereby diminishing the buffering effect of this protective factor and indirectly exacerbating bedtime procrastination. Based on the above reasoning, we propose the following hypothesis: Psychological resilience may mediate the relationship between social exclusion and bedtime procrastination.

Fear of missing out (FoMO) refers to a pervasive anxiety that individuals experience due to concerns about missing out on others’ novel experiences or positive events (Casale et al., 2018), and its core characteristic lies in the desire to remain continuously connected with what others are doing (Przybylski et al., 2013). This phenomenon is particularly prevalent among college students (Piko et al., 2025). According to basic psychological needs theory (BPNT) (Ryan and Deci, 2017), individuals possess innate basic psychological needs, which include the need for relatedness. The need for relatedness refers to individuals’ desire to establish intimate and warm connections with others and to experience a sense of belonging and being cared for. When the need for relatedness is chronically frustrated, individuals may exhibit compensatory behaviors or seek alternative sources of satisfaction (Sheldon et al., 2011).

Based on this, for individuals with high levels of FoMO, the need for relatedness is already in a state of chronic dissatisfaction. Over time, this need may become less salient, or even emotionally blunted in this group. Under such circumstances, social exclusion may merely serve to reaffirm their existing experience of unmet relational needs, rather than constitute a new or significant setback. Consequently, these individuals may no longer experience strong compensatory motivation in response to social exclusion and, thus, may be less likely to spend excessive pre-sleep time engaging in compensatory activities. Therefore, for individuals with high levels of FoMO, the association between social exclusion and bedtime procrastination may be weak or even non-significant.

In contrast, for individuals with relatively low levels of FoMO, the need for relatedness is generally in a relatively satisfied state. When they encounter social exclusion, this otherwise well-maintained need for relatedness becomes thwarted, thereby disrupting their previous psychological equilibrium and generating a strong sense of frustration. This, in turn, triggers intense compensatory motivation (Kwan et al., 2021), which may drive them to sacrifice nighttime rest for compensatory activities—such as engaging with social media or interpersonal interactions—to restore threatened relatedness. Such nighttime compensatory behaviors are precisely in conflict with going to bed on time, thereby potentially exacerbating bedtime procrastination (Kroese et al., 2014b). Accordingly, the present study proposes the following hypothesis: Fear of missing out may moderate the relationship between social exclusion and bedtime procrastination.

**Figure 1:**
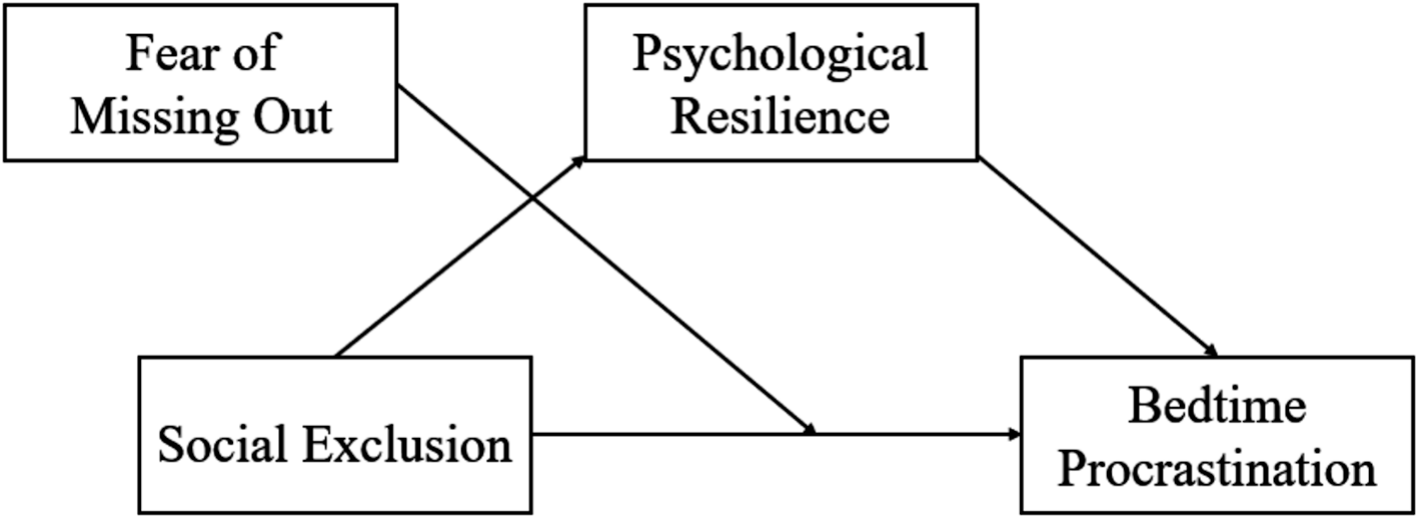
Theoretical model.

In summary, the present study integrates ego depletion theory (Baumeister et al., 1998), the stress-buffering model (Cohen and Wills, 1985), and BPNT (Ryan and Deci, 2017) to construct a multi-level theoretical explanatory framework. Specifically, ego depletion theory explains why social exclusion weakens individuals’ self-control resources, thereby triggering bedtime procrastination. However, this process is not a direct linear relationship; rather, individual psychological resource traits (e.g., psychological resilience) and affective traits (e.g., FoMO) play critical roles in this process. The stress-buffering model further suggests that psychological resilience is conceptualized as an important personal internal resource, which can be depleted by social exclusion, thereby exacerbating bedtime procrastination. Meanwhile, BPNT suggests that the effect of social exclusion on bedtime procrastination may vary depending on individuals’ levels of FoMO. By integrating the aforementioned theoretical frameworks, the present study develops a moderated mediation model to investigate the underlying mechanisms linking social exclusion and bedtime procrastination among college students. This study further examines the mediating role of psychological resilience and the moderating role of FoMO in the relationship between social exclusion and bedtime procrastination (see Figure 1 for the theoretical model).

Based on this, the following research hypotheses are proposed:

*H1*: Social exclusion is significantly and positively associated with bedtime procrastination among college students.

*H2*: Psychological resilience mediates the relationship between social exclusion and bedtime procrastination.

*H3*: Fear of missing out moderates the association between social exclusion and bedtime procrastination.

## Methods

### Participants and procedure

The commonly used formula for estimating the required sample size for a population proportion is as follows: N represents the required sample size; Z represents the critical value of the standard normal distribution, which is typically set at 1.96 for a 95% confidence level; p represents the expected population proportion, which is conservatively assumed to be 0.5 in the absence of prior information; and e represents the maximum allowable margin of error, which is typically set at ±5% in research (Cochran, 1977). Applying this formula yielded a minimum required sample size of 384.

$$N=\frac{Z^{2}*p(1-p)}{e^{2}}$$

In this study, a convenience sampling method was employed, and undergraduate students from a university in China were selected as participants. Data were collected between September 2025 and February 2026 using a self-administered questionnaire, which was designed and distributed via the online platform “Wenjuanxing.” A total of 1,050 valid questionnaires were initially collected. The following exclusion criteria were applied: (a) missing responses on any item, (b) identical answers for 10 or more consecutive items, (c) obvious patterned responding (e.g., a zigzag pattern), and (d) completion time of less than 1 min. Based on these criteria, 78 questionnaires were excluded, yielding 972 valid responses, with an effective response rate of 92.6%.

The demographic characteristics of the participants are presented in Table 1. Among the 972 respondents, the sex distribution was relatively balanced, with a slightly higher proportion of female participants (*n* = 571, 58.7%). The participants were drawn from four grades: freshmen (*n* = 611, 62.9%), sophomores (*n* = 286, 29.4%), juniors (n = 63, 6.5%), and graduating grade students (*n* = 12, 1.2%). The participants aged in the range of 17–25 years (*M* = 19.44, SD = 1.53). Regarding academic majors, the majority of participants were from science and engineering disciplines (71.7%), while the remainder were from humanities and social sciences (28.3%).

**Table 1:** Demographic characteristics of the sample (*N* = 972).

Variable	Category	Frequency (n)	Percentage (%)
Sex	Male	401	41.3
	Female	571	58.7
Grade	Freshman	611	62.9
	Sophomore	286	29.4
	Junior	63	6.5
	Graduating grade	12	1.2
Academic majors	Humanities and social sciences	275	28.3
	Science and engineering	697	71.7

### Measurement of variables

The primary variables measured in this study included social exclusion, bedtime procrastination, psychological resilience, and fear of missing out (FoMO). These measurement instruments have been demonstrated to possess good reliability and validity in previous empirical studies.

#### Social exclusion

The Social Exclusion Scale, developed by Wu H. et al. (2013), was used to measure the extent to which college students experience social exclusion events. The scale consists of 19 items and is divided into two dimensions: direct exclusion and indirect exclusion. All items are rated on a 5-point Likert scale (1 = “never” to 5 = “always”), with total scores ranging from 19 to 95. No reverse-scored items are included. The total score of the scale was used for analysis in this study, with higher scores indicating higher levels of social exclusion among college students. In this study, Cronbach’s *α* coefficient for this scale was 0.980.

#### Bedtime procrastination

Bedtime procrastination was assessed using the Chinese version of the Bedtime Procrastination Scale, which was originally developed by Kroese et al. (2014a) and adapted by Ma et al. (2021). The scale consists of nine items rated on a 5-point Likert scale (1 = “strongly disagree” to 5 = “strongly agree”), with total scores ranging from 9 to 45. A total of four items (Items 2, 3, 7, and 9) are reverse-scored. After reverse-scoring these items, the total score of the scale was used for analysis in this study, with higher scores indicating more severe bedtime procrastination. In this study, Cronbach’s α coefficient for this scale was 0.843.

#### Psychological resilience

The clinical measurement of individual psychological resilience was performed using the Chinese version of the Connor–Davidson Resilience Scale, originally developed jointly by Connor and Davidson and translated and revised by Yu and Zhang (2007), to assess individual psychological resilience in the general population. The scale has 25 items, including three dimensions: hardiness, optimism, and strength. A 5-point scoring method is used, where 1 represents “never,” 2 represents “rarely,” 3 represents “sometimes,” 4 represents “often,” and 5 represents “always.” The total score ranges from 25 to 125, with no reverse-scored items. In this study, the total score of this scale was used for analysis; the higher the total score, the higher the individual’s psychological resilience level. In this study, Cronbach’s *α* coefficient of this scale was 0.963.

#### Fear of missing out

The Fear of Missing Out Scale developed by Przybylski et al. (2013) was adopted. The Chinese version of the FoMO Scale was adapted by Chai et al. (2018) to reflect the Chinese local cultural context. The revised version consists of 10 items, using a 5-point Likert scale, with total scores ranging from 10 to 50 and no reverse-scored items. On the scale, 1 represents “never,” 2 represents “rarely,” 3 represents “sometimes,” 4 represents “often,” and 5 represents “always.” In this study, the total score of the scale was used for analysis, with higher total score indicating higher level of fear of missing out among college students. In this study, Cronbach’s α coefficient of this scale was 0.848.

### Data analysis

All statistical analyses were performed using SPSS version 27.0, and a *p* < 0.05 was considered statistically significant. Descriptive statistics were presented as frequencies (n) and percentages (%) for categorical variables and as mean ± standard deviation (M ± SD) for continuous variables.

In this study, Hayes’ PROCESS macro (Model 4 and Model 5) was used to test the hypothesized mediating and moderating effects. Specifically, the mediating effect of psychological resilience on the relationship between social exclusion and bedtime procrastination was first examined using the mediation analysis; subsequently, the moderating role of fear of missing out was tested using the moderation analysis.

## Results

### Test for common method bias

To assess potential common method bias in the data, Harman’s single-factor test was conducted using the principal component analysis on all measurement items. The results showed that eight factors had eigenvalues greater than 1, collectively accounting for 67.60% of the variance. The largest variance explained by the first factor was 30.93%, which is below the critical threshold of 40%, indicating that common method bias in this study was within an acceptable range.

### Analysis of demographic differences in study variables

#### Analysis of sex differences in study variables

Independent samples *t*-tests were conducted to examine sex differences in social exclusion, bedtime procrastination, psychological resilience, and fear of missing out. The results showed (see Table 2) that there was a significant sex difference in social exclusion among college students (*t* = 5.748, *p* < 0.001), with male college students scoring significantly higher than female college students. No significant sex differences were found in bedtime procrastination, psychological resilience, or fear of missing out among college students.

**Table 2:** Analysis of differences in study variables by sex (M ± SD).

Variable	Male (*n* = 401)	Female (*n* = 571)	*t*	*p*
1. Social exclusion	37.71 ± 17.18	31.54 ± 15.37	5.75	<0.001
2. Bedtime procrastination	26.07 ± 6.21	26.69 ± 6.44	−1.51	0.94
3. Psychological resilience	85.41 ± 18.64	87.00 ± 17.38	−1.37	0.34
4. Fear of missing out	27.54 ± 7.41	28.18 ± 6.70	−1.40	0.07

^*^p < 0.05, ^**^p < 0.01, ^***^p < 0.001.

#### Analysis of differences in study variables by grade

A one-way analysis of variance (ANOVA) was conducted to examine differences in social exclusion, bedtime procrastination, psychological resilience, and fear of missing out across grade. The results showed (see Table 3) that there were significant differences across grade in social exclusion among college students (*F* = 3.26, *p* < 0.05). The results of *post hoc* multiple comparisons indicated that social exclusion among graduating grade students was significantly higher than that among freshmen; bedtime procrastination was higher among juniors than among freshmen and sophomores; and there were no significant differences in psychological resilience and fear of missing out among college students across different grades.

**Table 3:** Analysis of differences in study variables by grade (M ± SD).

Grade	Social exclusion	Bedtime procrastination	Psychological resilience	Fear of missing out
① Freshmen (*n* = 611)	33.09 ± 15.96	26.23 ± 6.48	86.90 ± 17.83	28.26 ± 6.80
② Sophomores (*n* = 286)	35.30 ± 17.09	26.37 ± 6.24	85.55 ± 18.27	26.99 ± 7.32
③ Junior (*n* = 63)	36.27 ± 15.96	28.59 ± 5.17	86.52 ± 16.27	28.48 ± 7.66
④ Graduating grade (*n* = 12)	44.42 ± 20.66	27.17 ± 6.51	76.00 ± 20.64	29.75 ± 3.77
*F*	3.26	2.71	1.72	2.57
*p*	0.02	0.04	0.16	0.05
*LSD*	①<④	①<③, ②<③		

^*^p < 0.05, ^**^p < 0.01, ^***^p < 0.001.

#### Analysis of differences in study variables by academic majors

Independent samples *t*-tests were conducted to examine differences in social exclusion, bedtime procrastination, psychological resilience, and fear of missing out across academic majors. The results showed (see Table 4) that there was a significant difference in bedtime procrastination between academic majors among college students (*t* = 2.345, *p* < 0.05), with students in humanities and social sciences scoring significantly higher than those in science and engineering. No significant differences were found in social exclusion, psychological resilience, or fear of missing out among college students across academic majors.

**Table 4:** Analysis of differences in study variables by academic majors (M ± SD).

Variable	Humanities and social sciences (*n* = 275)	Science and engineering (*n* = 697)	*t*	*p*
1. Social exclusion	33.74 ± 17.13	34.22 ± 16.14	−0.41	0.35
2. Bedtime procrastination	27.19 ± 6.52	26.13 ± 6.26	2.30	0.02
3. Psychological resilience	85.21 ± 18.72	86.79 ± 17.59	−1.24	0.22
4. Fear of missing out	28.49 ± 7.44	27.69 ± 6.82	1.60	0.11

^*^p < 0.05, ^**^p < 0.01, ^***^p < 0.001.

### Correlation analysis

As shown in Table 5, social exclusion was significantly and negatively correlated with psychological resilience (*r* = −0.28, *p* < 0.01) and significantly and positively correlated with bedtime procrastination among college students (*r* = 0.23, *p* < 0.01). Psychological resilience was significantly and negatively correlated with bedtime procrastination (*r* = −0.33, *p* < 0.01). Fear of missing out was significantly and positively correlated with social exclusion (*r* = 0.29, *p* < 0.01) and bedtime procrastination (*r* = 0.37, *p* < 0.01) and significantly and negatively correlated with psychological resilience (*r* = −0.09, *p* < 0.01). In addition, correlation analysis found that sex was significantly and negatively correlated with social exclusion (*r* = −0.19, *p* < 0.01), academic major was significantly and negatively correlated with bedtime procrastination (*r* = −0.08, *p* < 0.05), and grade was significantly and positively correlated with social exclusion and bedtime procrastination (*r* = 0.07, 0.09, *p* < 0.05). Therefore, these three variables were included as control variables in subsequent analyses. Correlation analysis clarified the interrelationships among the variables, providing a basis for constructing the subsequent moderated mediation model.

**Table 5:** Correlations among study variables.

Variable	1	2	3	4	5	6	7
1. Sex	1						
2. Academic majors	−0.29^**^	1					
3. Grade	−0.08^*^	0.18^**^	1				
4. Bedtime procrastination	0.05	−0.08^*^	0.07^*^	1			
5. Social exclusion	−0.19^**^	0.01	0.09^**^	0.23^**^	1		
6. Psychological resilience	0.04	0.04	−0.05	−0.34^**^	−0.28^**^	1	
7. Fear of missing out	0.04	−0.05	−0.03	0.37^**^	0.29^**^	−0.09^**^	1

^*^p < 0.05, ^**^p < 0.01, ^***^p < 0.001.

### The moderated mediation model

Based on the correlation analysis, it was found that social exclusion was significantly correlated with sex and academic majors, and bedtime procrastination was significantly correlated with grade and academic majors. Therefore, sex, academic majors, and grade were included as control variables and were dummy-coded before the mediation effect test (see Table 6).

**Table 6:** Dummy coding of control variables.

Independent variables	Coding
Sex	
Sex	Female = 1 Male = 0
Grade	
Grade 1	freshmen = 1 sophomores = 0 junior = 0 graduating grade = 0
Grade 2	freshmen = 0 sophomores = 1 junior = 0 graduating grade = 0
Grade 3	freshmen = 0 sophomores = 0 junior = 1 graduating grade = 0
Academic	
Major	science and engineering = 1 humanities and social sciences = 0

^*^p < 0.05, ^**^p < 0.01, ^***^p < 0.001.

**Figure 2:**
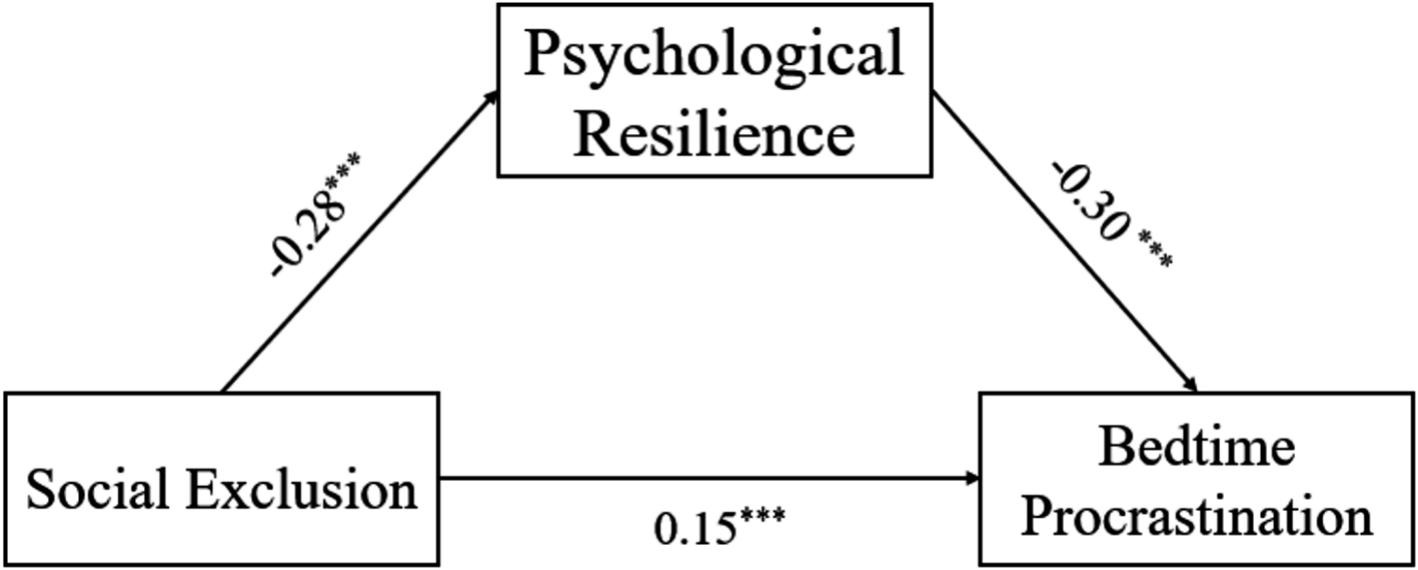
Mediation effect of psychological resilience on bedtime procrastination. ^***^p < 0.001.

After controlling for sex, grade, and major, bedtime procrastination was specified as the dependent variable, social exclusion as the independent variable, and psychological resilience as the mediating variable. All continuous variables were standardized, and the PROCESS macro (Model 4) in SPSS was used to test the mediating effect of psychological resilience on the relationship between social exclusion and bedtime procrastination. The results showed (see Table 7) that social exclusion significantly and positively predicted bedtime procrastination (*β* = 0.24, *p* < 0.001) and significantly and negatively predicted psychological resilience (*β* = −0.28, *p* < 0.001). After including the mediating variable, social exclusion significantly and positively predicted bedtime procrastination (*β* = 0.15, *p* < 0.001), and psychological resilience significantly and negatively predicted bedtime procrastination (*β* = −0.30, *p* < 0.001). The mediating effect value of psychological resilience was 0.08, with a confidence interval of [0.06, 0.12], accounting for 35.42% of the total effect (see Table 8). These findings indicate that psychological resilience plays a partial mediating role in the relationship between social exclusion and bedtime procrastination, as shown in Figure 2.

**Table 7:** Mediation effect analysis of psychological resilience between social exclusion and bedtime procrastination.

Outcome variable	Predictor variable	*R*	*R* ^2^	*F*	*β*	*t*
Bedtime procrastination		0.27	0.07	12.15^***^		
	Sex 1				0.08	2.29
	Grade1				0.02	0.11
	Grade 2				0.03	0.24
	Grade 3				0.10	1.33
	Major1				−0.07	−2.06
	Social exclusion				0.24	7.44^***^
Psychological resilience		0.29	0.08	14.64^***^		
	Sex 1				0.01	0.84
	Grade1				0.21	0.13
	Grade 2				0.17	0.18
	Grade 3				0.11	0.15
	Major1				0.05	0.14
	Social exclusion				−0.28	−8.74^***^
Bedtime procrastination		0.39	0.15	25.24^***^		
	Sex 1				0.08	2.46
	Grade1				0.08	0.60
	Grade 2				0.08	0.67
	Grade 3				0.13	1.85
	Major1				−0.05	−1.69
	Psychological resilience				−0.30	−9.83^***^
	Social exclusion				0.15	4.85^***^

^*^p < 0.05, ^**^p < 0.01, ^***^p < 0.001.

**Table 8:** Decomposition of total, direct, and mediating effects.

Effect type	Effect value	Boot SE	Boot CI	Relative effect size
Total effect	0.23	0.03	[0.17,0.29]	
Direct effect	0.15	0.03	[0.09,0.21]	64.58%
Indirect effect	0.08	0.02	[0.06,0.12]	35.42%

^*^p < 0.05, ^**^p < 0.01, ^***^p < 0.001.

**Figure 3:**
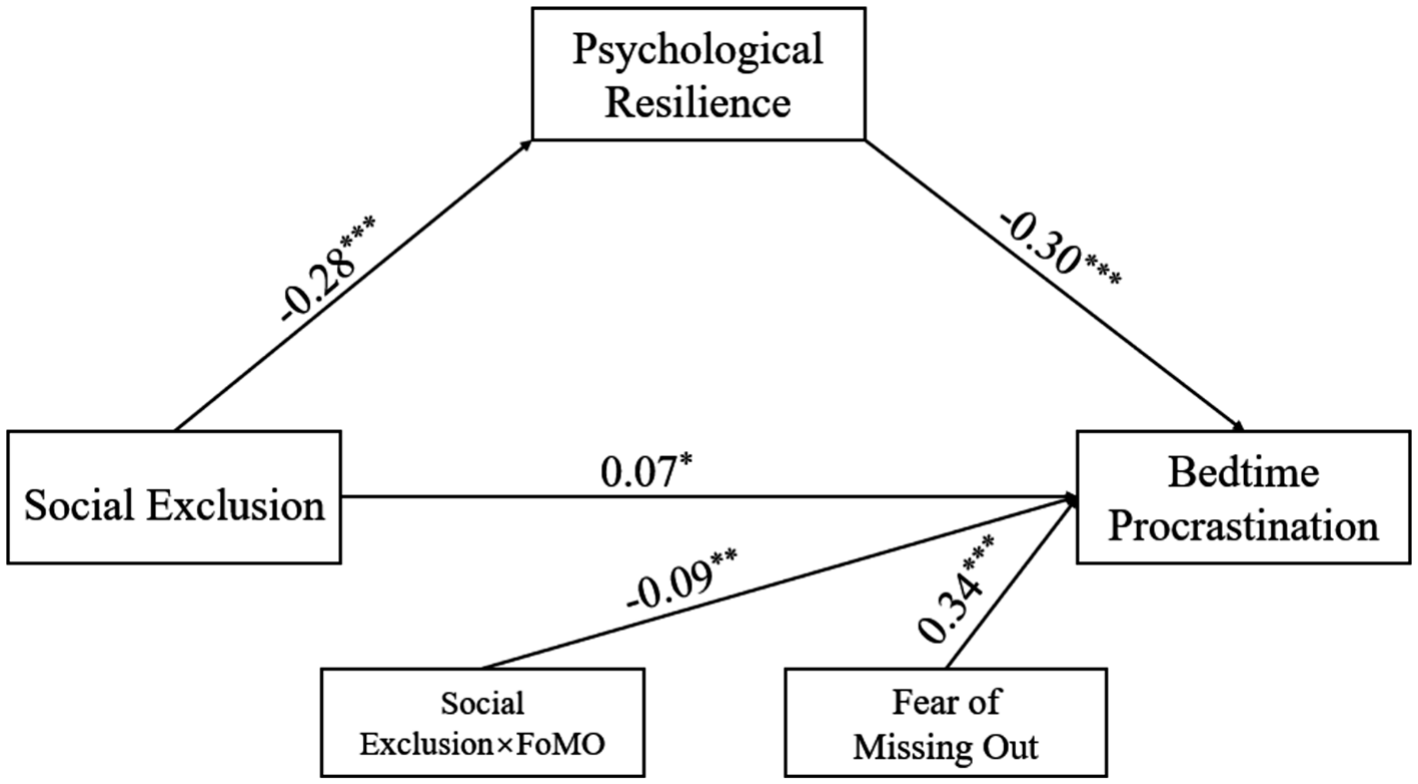
The moderated mediation model (*n* = 972). ^*^p < 0.05, ^**^p < 0.01, ^***^p < 0.001.

A total of two models were constructed in this study to evaluate the moderated mediating roles of psychological resilience and fear of missing out on the relationship between social exclusion and bedtime procrastination among college students (see Table 9 and Figure 3). The results showed that, in Model 1, the predictive effect of social exclusion on psychological resilience was significant (*β* = −0.28, *t* = −9.11, *p* < 0.001), whereas psychological resilience was also a significant predictor of bedtime procrastination among college students (*β* = −0.30, *t* = −10.27, *p* < 0.001), indicating that psychological resilience plays a mediating role between social exclusion and bedtime procrastination. The interaction term between social exclusion and fear of missing out had a significant effect on bedtime procrastination among college students (*β* = −0.09, *t* = −3.57, *p* < 0.01), indicating that fear of missing out plays a moderating role in the effect of social exclusion on bedtime procrastination among college students.

**Table 9:** Moderated mediation model of psychological resilience and fear of missing out in the relationship between social exclusion and bedtime procrastination among college students (*n* = 972).

Variables	Model 1 (outcome variable: psychological resilience)			Model 2 (outcome variable: bed procrastination)		
	*β*	SE	*t*	*β*	SE	*t*
Sex	0.01	0.07	0.20	0.10	0.06	1.60
Grade1	0.43	0.28	1.53	0.14	0.25	0.54
Grade 2	0.38	0.28	1.33	0.23	0.26	0.90
Grade 3	0.44	0.30	1.44	0.53	0.27	1.96
Academic majors	0.11	0.07	1.49	−0.13	0.07	−1.94
Social exclusion	−0.28	0.03	−9.11^***^	0.07	0.03	2.41^*^
Psychological resilience				−0.30	0.03	−10.27^***^
FoMO				0.34	0.03	11.50^***^
Social exclusion × FoMO				−0.09	0.02	−3.57^**^
*R* ^2^	0.08			0.26		
*F*	14.64^***^			37.83^***^		

^*^p < 0.05, ^**^p < 0.01, ^***^p < 0.001.

**Figure 4:**
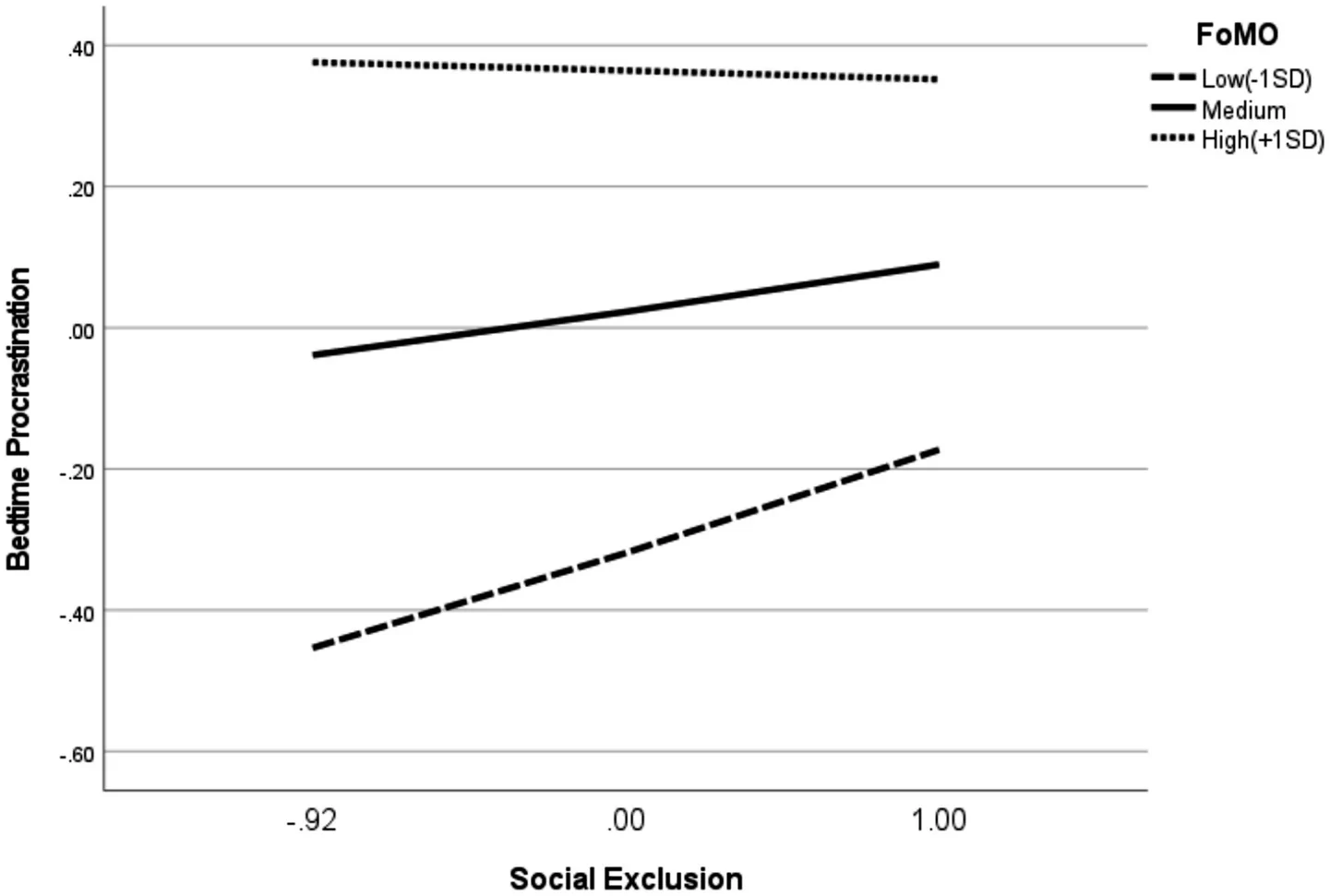
The moderated effect of FoMO on the relationship between social exclusion and bedtime procrastination.

The analysis of the mediating effect of psychological resilience on bedtime procrastination among college students showed that the direct effect of psychological resilience on bedtime procrastination among college students was −0.30, with a 95% confidence interval of [−0.35, −0.24]. The specific effect values and confidence intervals for the effect of social exclusion on bedtime procrastination among students with different levels of fear of missing out are detailed in Table 10. To further explore the moderating effect of fear of missing out on the relationship between social exclusion and bedtime procrastination among college students, the simple slope analysis method was adopted to examine the effect of social exclusion on bedtime procrastination among college students at different levels of fear of missing out (see Figure 4 for details). The results showed that, among college students with low and moderate levels of fear of missing out, the positive effect of social exclusion on bedtime procrastination was significant. Specifically, within a certain range, as the level of fear of missing out increased, the effect of social exclusion on bedtime procrastination weakened; however, among college students with high levels of fear of missing out, this path was not significant.

**Table 10:** Moderating effect of FoMO on the relationship between social exclusion and bedtime procrastination (*n* = 972).

FoMO	Effect	Boot SE	BootLLCI	BootULCL
Low	0.16	0.04	0.08	0.25
Medium	0.08	0.03	0.01	0.14
High	−0.01	0.04	−0.08	0.06

^*^p < 0.05, ^**^p < 0.01, ^***^p < 0.001.

## Discussion

The results of this study supported the validity of the moderated mediation model, revealing that there was a significant positive correlation between social exclusion and bedtime procrastination among college students, that psychological resilience played a mediating role in the relationship between social exclusion and bedtime procrastination, and that the direct path in this mediation model was significantly moderated by the level of fear of missing out. These findings deepen our understanding of the formation mechanisms of bedtime procrastination, integrating bedtime procrastination, social exclusion, psychological resilience, and fear of missing out into a unified framework, and provide a theoretical basis and practical guidance for future interventions targeting college students’ sleep health.

### Discussion on demographic variables

This study found that social exclusion among college students was influenced by sex, with male college students experiencing higher levels of social exclusion than female college students. This sex difference may stem from the relative advantages of female individuals in interpersonal skills (Vázquez et al., 2025). Specifically, female individuals typically possess higher levels of social skills (Hajovsky et al., 2022). They are more emotionally sensitive and exhibit greater empathy, enabling them to more keenly perceive others’ emotional states and interaction details, thereby adjusting the interaction atmosphere in a timely manner and adopting more flexible coping strategies (OECD, 2024; Lee et al., 2025). In addition, female individuals tend to have more proactive and positive interaction attitudes. Their verbal expression and emotional communication abilities are stronger, which makes their interpersonal relationships generally more optimistic and harmonious than those of male individuals (Tang et al., 2025), thereby placing them at a relatively lower risk of social exclusion.

In addition, this study showed that both social exclusion and bedtime procrastination among college students exhibited significant differences by grade. Regarding social exclusion, the level of social exclusion among graduating grade students was significantly higher compared to freshmen students. This may be closely related to the employment pressure faced by graduating grade students. Currently, college graduates are encountering an increasingly challenging employment situation, with employment pressure continuing to rise (Li, 2023). In the process of job hunting, limited job resources make the competition among graduating grade students increasingly fierce, and the competitive environment on campus itself can exacerbate the occurrence of social exclusion behaviors (Zhang et al., 2024). Meanwhile, compared to freshmen, graduating students may receive less attention and support from their teachers, leaving them with insufficient social support when facing employment-related difficulties. Therefore, senior students in the context of employment competition may be more likely to experience higher levels of social exclusion.

Regarding bedtime procrastination, it was found that junior students exhibited significantly higher levels than freshmen and sophomores. As the grade level increases, the academic pressure faced by college students generally increases. Junior students are at a critical stage of their academic development and often need to balance multiple tasks simultaneously, such as thesis writing, internship preparation, career planning, and preparation for postgraduate entrance examinations. These tasks are not only time-consuming and labor-intensive but also associated with considerable pressure. To cope with the above pressures, students may tend to sacrifice sleep time to complete academic tasks or other responsibilities, thereby leading to bedtime procrastination. The increasing number of problems faced by junior students may lead to various negative emotions, and the generation of such negative emotions may also affect individuals’ bedtime (Zhu et al., 2022), resulting in bedtime procrastination.

In this study, freshmen accounted for 62.9% of the sample, while senior students accounted for only 7.7%. Considering the compositional characteristics of the participant sample, freshmen are in the transitional period from high school to college, during which their peer support networks have not yet been fully established. This unique developmental characteristics may impact their perception of social exclusion (Arnett, 2000). Previous longitudinal studies have found that the level of social exclusion among freshmen is relatively high upon entering college and then shows a declining trend, and psychological resilience can accelerate this decline (Ge et al., 2025). Therefore, the pathway of “social exclusion → psychological resilience → bedtime procrastination” revealed in this study may be particularly pronounced among freshmen, and the moderating effect of FoMO may also partially reflect freshmen’s unique adaptation patterns—that is, freshmen who are highly concerned about social connections may become habituated due to repeated exposure, thereby weakening the actual impact of social exclusion. Due to the small number of senior students in the sample, the results of this study primarily reflect the characteristics of freshmen, and generalization to other grade levels or populations requires caution. Future research should examine the applicability of this model across different academic stages.

Finally, college students of different majors also showed significant differences in bedtime procrastination, with students in the humanities and social sciences scoring significantly lower than those in science and engineering. This result can be reasonably explained by real-world observations, which may stem from differences in task structures and daily schedules between the two disciplines. Laboratory work and exercises in science and engineering typically have clear time limits and fixed venues for completion, forming a strong external constraint mechanism. In contrast, learning in the humanities and social sciences primarily involves reading and writing, with more flexible but vague task boundaries, relying more on individual self-planning, which may easily lead to the continuous postponement of bedtime. Meanwhile, learning in the humanities and social sciences is often accompanied by active nighttime thinking and contemplative tendencies, and coupled with more flexible daytime schedules, these factors may also collectively contribute to bedtime procrastination.

### Social exclusion and college students’ bedtime procrastination

The results of this study showed that social exclusion was significantly and positively correlated with bedtime procrastination among college students; that is, the higher the level of social exclusion experienced by college students, the more severe their bedtime procrastination. This finding is highly consistent with existing empirical evidence. For example, previous studies have found that social exclusion is significantly and positively correlated with sleep quality among college students (Liu et al., 2023), and bedtime procrastination has been considered one of the important behavioral pathways potentially contributing to decreased sleep quality. Bedtime procrastination is essentially a form of self-regulation failure, manifested as the unnecessary and voluntary delay of bedtime in the absence of external constraints (Kamphorst et al., 2018). Self-control ability is significantly and negatively correlated with bedtime procrastination; individuals with low self-control find it more difficult to adhere to their intended bedtime (Vazsonyi et al., 2025). Social exclusion, in turn, has been theorized as a typical context for the depletion of self-regulation resources. The temporal need-threat model, proposed by psychologist Williams (2009), offers a useful framework for understanding individuals’ psychological responses after experiencing social exclusion. The core proposition of this model is that social exclusion threatens four fundamental psychological needs—belongingness, self-esteem, control, and meaningful existence—and that this threat unfolds across three dynamic stages over time. According to this model (Cheng et al., 2011), social exclusion may severely impair individuals’ basic need for belonging, potentially triggering the social pain and psychological distress characteristic of exclusion, which may, in turn, lead individuals to invest substantial cognitive and emotional resources to cope with the negative feelings associated with exclusion (Peng and Tao, 2019). Individuals who experience social exclusion often exhibit a general decline in self-regulatory ability. It is plausible that, when individuals have expended significant psychological resources during the day to cope with the anxiety, loneliness, and frustrated belongingness needs often linked to social exclusion, their self-control resources may become relatively depleted by nighttime, which could, in turn, make it difficult to resist the temptation of immediate gratification, such as continuing to scroll through mobile phones or watching short videos. This process might help explain why they tend to deviate from their bedtime intentions and report significant bedtime procrastination.

### The mediating effect of psychological resilience

The results of this study reported that social exclusion showed a significant negative association with psychological resilience and that psychological resilience was also significantly and negatively associated with bedtime procrastination among college students, indicating that psychological resilience mediated the relationship between social exclusion and bedtime procrastination. This finding is consistent with the interpretation that social exclusion may be associated with severe bedtime procrastination not only directly but also indirectly through its negative relationship with individuals’ psychological resilience.

The conservation of resources theory, proposed by Hobfoll (1989), posits that individuals are motivated to obtain, maintain, and protect the resources they value and that psychological stress primarily stems from actual or potential loss of these resources. Psychological resilience, as a key internal resource that helps individuals effectively cope with adversity and recover from stress, is believed to play an important buffering role in individuals’ responses to environmental challenges (Schäfer et al., 2023). According to the resource depletion logic of this theory, social exclusion, as a threatening social stressor (Lutz et al., 2024), is associated with lower levels of psychological resilience, which may reflect the depletion of internal resources required for individuals to maintain mental health. In the context of social exclusion, individuals may experience persistent negative emotions and psychological defensive states due to frustrated belongingness needs and damaged social relationships and may be required to continuously mobilize and expend psychological resilience resources to cope with the resulting discomfort (Clercq and Pereira, 2024; Rudert and Mühlbauer, 2024). Therefore, social exclusion, through its potential to continuously consume individuals’ psychological resilience resources, may contribute to a significant decline in their psychological resilience levels, thereby potentially weakening their internal capacity to cope with subsequent stress and engage in self-regulation.

On the other hand, psychological resilience showed a significant negative association with bedtime procrastination and may serve to buffer the risk of bedtime procrastination. The conservation of resources theory also suggests that individuals with sufficient psychological resilience resources may be more likely to effectively regulate their behaviors in the face of evening temptations and conflicts, maintain regular bedtime routines, and reduce bedtime procrastination. Empirical evidence has provided support for this notion. Huang et al. (2025) found that psychological resilience is significantly and negatively correlated with bedtime procrastination among Chinese college students. College students with lower psychological resilience, due to insufficient psychological resource reserves, may be more prone to self-regulation failure when faced with temptations of immediate gratification in the evening, such as scrolling through mobile phones or watching videos, which could, in turn, make it more difficult to adhere to their intended bedtime and thus lead to more severe bedtime procrastination.

Therefore, in interventions targeting college students’ bedtime procrastination, universities should not only consider reducing the occurrence of social exclusion but also pay attention to enhancing students’ psychological resilience levels and fostering positive psychological resources to potentially mitigate the negative effects of social exclusion on sleep behaviors.

### The moderating effect of fear of missing out

In the results of the moderating effect, it is interesting to note that, among college students with low and moderate levels of fear of missing out, the positive predictive effect of social exclusion on bedtime procrastination was significant; however, among college students with high levels of fear of missing out, this effect was no longer significant. This finding further extends the theoretical model of the relationship between social exclusion and bedtime procrastination from the perspective of moderating effects.

This study also found that, within a certain range, as the level of fear of missing out increased, the effect of social exclusion on bedtime procrastination weakened. This result can be explained from the perspective of BPNT. This theory posits that individuals have a basic psychological need for relatedness, and when this need is chronically unsatisfied, individuals’ behavioral responses undergo a dynamic progression from compensatory reactions to alternative satisfaction and then to numbing (Ryan and Deci, 2017).

For individuals with low levels of fear of missing out, their daily need for relatedness is in a relatively satisfied state (Servidio et al., 2024), and they lack sufficient experience in coping with social exclusion. When they experience social exclusion, their need for relatedness is suddenly thwarted, and this group finds it difficult to quickly initiate compensatory social behaviors to repair the damaged psychological needs, causing their psychological tension to persist into the nighttime. This may manifest as being forced to prolong wakefulness before sleep to compensate by seeking social connections, which constitutes bedtime procrastination.

For individuals with high levels of fear of missing out, they are in a chronic state of unsatisfied relatedness needs (Xie et al., 2018). According to the core assumption of BPNT, when this basic need is continuously thwarted to a certain extent, individuals may activate a numbing mechanism, actively downregulating their sensitivity to this need to avoid repeated experiences of disappointment and pain. Meanwhile, they may have already achieved alternative satisfaction of their needs through non-social activities (e.g., short videos, games, etc.) and may no longer rely on social behaviors as the primary means of psychological satisfaction, thereby rendering the effect of social exclusion on bedtime procrastination non-significant.

### Implications

At the theoretical level, this study not only revealed the direct association between social exclusion and bedtime procrastination among college students but also elucidated the mediating and moderating roles of psychological resilience and fear of missing out in the relationship between social exclusion and bedtime procrastination, achieving the integration of multiple theoretical models. At the practical level, the findings of this study provide a basis for formulating strategies to prevent and cope with social exclusion, which may help reduce bedtime procrastination among college students. According to the latest trends in health behavior change interventions (Tarkhan and Shabani, 2023), behavioral interventions targeting bedtime procrastination should focus on the following aspects. First, individuals’ emotion regulation abilities should be enhanced, particularly by training in adaptive strategies such as cognitive reappraisal, to reduce reliance on avoidant strategies. Second, physical activity, such as moderate-intensity aerobic exercise during the daytime, should be incorporated to improve overall emotional state and sleep quality. It is worth emphasizing that, even after partial sleep deprivation, low-intensity aerobic exercise can still help maintain a certain level of cognitive performance (Monleon et al., 2023), suggesting that physical activity may partially offset the risk of cognitive decline caused by bedtime procrastination and providing empirical evidence for college students to adopt remedial exercise after experiencing sleep insufficiency. Third, targeted pre-sleep behavioral guidelines should be developed (e.g., limiting electronic device use and establishing relaxing bedtime routines) to help individuals break the vicious cycle of “staying up late scrolling through mobile phones.” Future research could conduct randomized controlled trials based on these directions to verify the effectiveness of interventions.

The above findings suggest that, in intervention efforts targeting college students’ sleep health, in addition to addressing the effects of social exclusion itself, attention should also be paid to distinguishing the psychological resource status of groups with different levels of fear of missing out, and differentiated psychological resource strategies should be adopted.

## Conclusion

This study constructed and tested a moderated mediation model to explore how social exclusion affects bedtime procrastination among college students. The results showed that social exclusion was significantly and negatively correlated with psychological resilience and significantly and positively correlated with bedtime procrastination, psychological resilience was significantly and negatively correlated with bedtime procrastination, and FoMO was significantly and positively correlated with both social exclusion and bedtime procrastination and significantly and negatively correlated with psychological resilience. The study also found that psychological resilience played a mediating role in the effect of social exclusion on bedtime procrastination, while FoMO exerted a moderating effect. These findings have important implications for improving bedtime procrastination among Chinese college students. Specifically, when intervening in college students’ bedtime procrastination, in light of the mediating role of psychological resilience, efforts should be made to enhance psychological resilience as a core protective resource, helping students buffer the depletion of self-control resources caused by social exclusion. Furthermore, interventions targeting bedtime procrastination among college students should fully consider the influence of different levels of FoMO. Future research may further integrate the above intervention elements to construct multi-level, systematic prevention and intervention programs for bedtime procrastination among college students.

### Limitations and future directions

This study has the following limitations: First, the cross-sectional design cannot establish causal relationships among the variables, and the directional pathways of the variables need further verification. Second, the sample was only drawn from a single university using convenience sampling, with a relatively high proportion of freshmen and science and engineering majors, limiting the generalizability of the findings to broader populations. Third, although the Harman single-factor test was used to assess common method bias, this test has limited power for data in which all variables are collected via self-report from the same participants at a single timepoint. Future research should adopt more rigorous statistical control methods, such as the common latent factor method. Fourth, to address the potential concern that the measurement of bedtime procrastination might have been subject to a ceiling effect among individuals in the high-FoMO group, we conducted supplementary analyses to examine this possibility, and the results indicated that this effect was not significant in our sample. Fifth, self-regulation was not directly measured. Although it was proposed on theoretical grounds that psychological resilience influences bedtime procrastination through self-regulation failure, no specific measurement tool was included in the study design. Therefore, the explanation regarding the mediating mechanism of self-regulation is primarily based on theoretical inference and lacks direct empirical testing. Finally, socioeconomic background information of the participants was not systematically collected. To address the above limitations, future research could adopt longitudinal tracking or experimental designs, expand the sampling scope to enhance generalizability, and introduce multi-method and multi-source data with more rigorous controls for common method bias. Future research should also include the measurement of self-regulation and employ experience sampling methods or experimental paradigms to dynamically track changes in self-regulation resources, thereby more rigorously testing the mediating pathway proposed in this study, while incorporating variables such as socioeconomic status as covariates in the model.

## Data Availability

The raw data supporting the conclusions of this article will be made available by the authors, without undue reservation.
