# Correction: The effect of social exclusion on bedtime procrastination among college students: the mediating role of psychological resilience and the moderating role of fear of missing out

**DOI:** 10.3389/fpsyg.2026.1994448

**Published:** 2026-09-23

**Authors:** Jianbo Wang, Zixuan Zhang, Jiale Zhao

**Affiliations:** School of Psychology and Mental Health, North China University of Science and Technology, Tangshan, China

**Keywords:** bedtime procrastination, college students, fear of missing out, psychological resilience, social exclusion

Author Zixuan Zhang was erroneously included as corresponding author.

The correct corresponding authors are Jianbo Wang and Jiale Zhao.

The original version of this article has been updated.

